# Effects of Ivy (*Hedera helix*) Leaf Extract on Growth, Digestive Enzyme Activity, Hematological Parameters, Innate Immunity, and the Expression of Immune-Related Genes in Rainbow Trout (*Oncorhynchus mykiss*)

**DOI:** 10.1155/anu/4030680

**Published:** 2025-07-22

**Authors:** Raha Fadaei, Ahmad Noori, Arash Akbarzadeh, Seyed Hossein Hoseinifar, Marina Paolucci

**Affiliations:** ^1^Department of Fisheries, Faculty of Marine Science and Technology, University of Hormozgan, Bandar Abbas, Iran; ^2^Department of Fisheries, Faculty of Fisheries and Environmental Sciences, Gorgan University of Agricultural Sciences and Natural Resources, Gorgan, Iran; ^3^Department of Science and Technologies, University of Sannio, Benevento 82100, Italy

**Keywords:** antioxidative status, gene expression, immunity, Ivy leaf extract, rainbow trout

## Abstract

Herbal bioactive compounds are effective in enhancing the antioxidant and immune status of fish, as they effectively neutralize oxidative stress. The present study examined the effectiveness of Ivy (*Hedera helix*) leaf extract (ILE) on growth performance, immune responses, antioxidant parameters, and the expression of immune-related genes in rainbow trout (*Oncorhynchus mykiss*). Four test diets were prepared as follows: a control diet at 0 mg of ILE (ILE0) and diets of 100 mg (ILE100), 150 mg (ILE150), and 200 mg (ILE200) of ILE/kg. A total number of 240 rainbow trout, with an initial weight of 6.38 ± 0.30 g, were divided into 12 tanks and fed on the experimental diets for 8 weeks. Results indicated that ILE150 and ILE200 exhibited significantly higher final weight (FW), weight gain, and specific growth rate compared to the control and ILE100 treatments. Additionally, fish fed with ILE showed significantly higher activity of digestive enzymes, including trypsin, pepsin, proteases, lipase, and amylase compared to the control group. The experimental diets also significantly affected hematological indices, including erythrocytes, leukocytes, hematocrit, hemoglobin, mean corpuscular volume (MCV), mean corpuscular hemoglobin concentration, and mean corpuscular hemoglobin, and their highest levels were observed in ILE150 and ILE200. Furthermore, a notable reduction was observed in aspartate aminotransferase (AST), alanine transaminase (ALT), and alkaline phosphatase (ALP) activities in all ILE-supplemented diets. Serum antioxidant enzymes, including catalase (CAT), glutathione peroxidase (GPX), and superoxide dismutase (SOD), significantly increased in ILE150 and ILE200, but serum malondialdehyde (MDA) levels significantly declined in treatments fed with ILE compared to the control group. Alternative complement, total immunoglobulin, total protein, and lysozyme levels were significantly higher in ILE-supplemented-diet treatments. The results also showed a significant upregulation of immune-related genes, such as interleukin 1β, interleukin-6, interleukin-8, and lysozyme, in ILE150 and ILE200 compared to the control and ILE100. In conclusion, administering ILE at doses of 150–200 mg/kg diet could improve the growth performance and immune-antioxidant capacity of rainbow trout.

## 1. Introduction

Polyphenols, or polyphenolic compounds, are a major class of phytochemicals from plants. As secondary metabolites, they have diverse phenolic structures and chemical compositions [1]. There are two main groups of polyphenols called nonflavonoids and flavonoids based on the number of aromatic groups with aliphatic carbon skeletons and phenols that connect these rings [2]. The physiological characteristics of polyphenols are primarily impacted by several factors, such as their chemical composition, interactions with other large molecules within plants, and their potential for quick absorption from the intestines [3].

A considerable number of the advantages, including antioxidant characteristics [4], anti-inflammatory properties [5], and growth enhancer [6], make herbal bioactive compounds, a valuable addition to aquaculture practices for improving the health and productivity of aquatic species. Polyphenolic compounds are regarded as valid alternatives to chemicals and antibiotics in aquaculture [7]. Studies indicates that these bioactive compounds enhance the antioxidant capacity of fish, helping to mitigate oxidative damage caused by environmental stressors [2, 8–10]. Additionally, they can modulate immune responses, leading to increased resistance against pathogens [11–13]. Studies have shown that incorporating polyphenol-rich diets can significantly improve growth and reproduction in various fish species, making them a valuable component of sustainable aquaculture practices [14]. The beneficial effects of dietary polyphenols or polyphenol-rich diets on antioxidant defenses, immune response, disease resistance, reproductive performance, and growth have been demonstrated in numerous fish species [15–17].

Rainbow trout (*Oncorhynchus mykiss*) is one of the most widely cultured freshwater fish species worldwide, valued for its rapid growth, adaptability, and high nutritional quality. It plays a significant role in global aquaculture, with production exceeding 1.1 million tonnes annually [18]. This species contributes substantially to the economy and aquaculture output of many countries. Notably, in 2023, Iran produced approximately 25% of the total global rainbow trout production, ranking as the world's leading producer [18]. Due to its preference for cold, well-oxygenated waters and efficient feed conversion, rainbow trout is extensively farmed using various systems, including open water net pens and land-based facilities. Its importance in aquaculture research is underscored by its biological characteristics, such as growth performance, digestion, and immune responses, making it a key species for studies aimed at improving fish health and production efficiency.

Ivy (*Hedera helix*) is a highly esteemed species belonging to the Araliaceae family [19]. The leaves of this plant are often utilized as the principal active component in numerous herbal concoctions [20]. Chemical analysis of *H. helix* leaves has revealed a diverse range of phytochemicals with remedial properties, including sterols, tannins, terpenoids, glycosides, phenols, alkaloids, flavonoids, saponins, volatile and fixed oils, vitamins, carbohydrates, reducing sugars, and minerals [20, 21], while various ethanolic extracts from the leaves, flowers, and both immature and ripe fruits have demonstrated notable antioxidant and antimicrobial activities [22–24]. Some studies have provided clear evidence that extracts from the flowers and fruits of ivy contain compounds capable of combating fungal infections. These extracts demonstrated notable antioxidant and antimicrobial activities, indicating their potential biological relevance in managing pathogenic fungi [24].

Despite its importance for human health [20], there have been no documented efforts to test Ivy products in the aquaculture industry, to the best of our knowledge. Therefore, the aim of this trial was to investigate Ivy leaf extract (ILE) and their impacts on the growth performance, immunity, and antioxidant parameters of rainbow trout (*Oncorhynchus mykiss*).

## 2. Materials and Methods

### 2.1. Ivy Polyphenols

Ivy leaf (*H. helix*) extract was provided by EPO Istituto Farmochimico Fitoterapico S.r.l., Via Stadera 19-20141 Milano, Italy. According to the producer, the ivy leaves were harvested from the wild during the summer. The extract was obtained from the collected leaves using ethanol/water. This extract, in powder form with a particle size of 300 µm, contained triterpenic saponins (hederacoside C, alpha-hederin, hederacoside B, and beta-hederin), flavonoids (quercetin and kaempferol), caffeilchinic acid, and sterols. The nutritional assessment of the extract indicated the following composition: 90%–95% carbohydrates, 0%–1% fat, 0%–1% protein, and 3%–5% minerals, with an energy value of 409 kcal/100 g.

### 2.2. Diets Preparation

A basal diet (Table 1) was formulated following the guidelines presented in [25], to serve as the control with no inclusion of ILE, while experimental diets were prepared by adding ILE at concentrations of 100 mg (ILE100), 150 mg (ILE150), and 200 mg (ILE200) of ILE/kg of feed. The dietary components were gently homogenized and then mixed with water (30%–40%). The dough was ground using an electric meat grinder (MG1400R, Pars Khazar, Tehran, Iran) and then formed into pellets tailored to the fish's mouth. These pellets underwent a meticulous air-drying process and were subsequently preserved at a temperature of −20°C until use in this study.

### 2.3. Rearing Condition and Experimental Design

This research was conducted at a regional fish farm located in Gorgan, Golestan Province, Iran. A total of 240 rainbow trout with initial weight of 6.88 ± 0.21 g were transferred into 12 1000 L tanks (each tank containing 20 fish). The fish underwent a 14-day acclimatization period in experimental tanks and were fed with a prepared basal diet three times a day (at 9:00 am, 12:00 pm, and 3:00 pm). After this acclimatization phase, fish in each experimental group were fed with the respective prepared experimental diets at a rate of 3% of body weight three times a day (at 9:00 am, 12:00 pm, and 3:00 pm) for a duration of 8 weeks. Throughout the experimental period, water quality parameters, including water temperature (13.2 ± 1°C), pH (7.9 ± 0.4), and dissolved oxygen (8 ± 0.43 mg/L), were routinely monitored to ensure optimal conditions for the fish.

### 2.4. Growth Indices

After the 8-week feeding trial, the fish were withheld from feed for 24 h prior to sampling. For sampling, fish were anaesthetized using 0.2 mL/L phenoxyethanol following the methodology outlined by Giannenas et al. [27] and subsequently were measured for the final weight (FW) and length. The assessment of growth performance and survival rate followed the calculations outlined below [28]:

  $$\begin{array}{c} \text{Weight gain}:\text{WG (g)}=\text{Final body weight}-\text{initial body weight}, \end{array}$$

  $$\begin{array}{c} \text{Survival rate}:\text{SR }\left(\%\right)=\left(\frac{\text{Final number of fish}}{\text{Initial number of fish}}\right)\times 100, \end{array}$$

  $$\begin{array}{c} \text{Specific growth rate}:\text{SGR}\left(\%\text{per day}\right)=\left(\frac{Ln\text{ final body weight}-Ln\text{ initial body weight}}{\text{Days}}\right)\times 100, \end{array}$$

  $$\begin{array}{c} \text{Feed conversion ratio}:\text{ FCR}=\left(\frac{\text{Final feed intake}}{\text{Final body weight}-\text{initial body weight}}\right). \end{array}$$

### 2.5. Digestive Enzymes Activities

The intestines and stomachs of four fish from each tank were dissected and rinsed with phosphate-buffered saline (PBS). Then, the tissue samples were separately homogenized in a Tris–HCl buffer (2 mL, 50 mM, pH 7.0). The suspension was centrifuged at 5000 × *g* at a temperature of 4°C for 10 min and the supernatant was separated for further digestive enzyme activity analysis. Bradford's method using bovine serum albumin solution as a standard solution was used to measure total soluble protein. Briefly, the protein reagent was prepared by mixing 0.01% (w/v) Coomassie brilliant blue G-250, 4.7% (w/v) ethanol, and 8.5% (w/v) phosphoric acid. Then, 5 mL of this reagent was added to the samples, and the absorbance was measured at 595 nm after 2 min and within 1 h using cuvettes. A reagent blank was prepared by mixing 0.1 mL of the appropriate buffer with 5 mL of the protein reagent for baseline correction [29]. Elisa kits were utilized for the quantification of trypsin (Cat. No.: CK-E91540, Eastbiopharm), chymotrypsin (Cat. No: CK-E92024, Eastbiopharm), and pepsin (Cat. No.: CK-E91413, Eastbiopharm) activities. The activity of amylase was measured by the Bernfeld [30] method. Briefly, the homogenate of the fish intestine was mixed with soluble starch and subjected to an incubation period of 30 min at a temperature of 37°C. To stop the reaction, dinitrosalicylic acid was introduced, followed by boiling for 5 min. Subsequently, distilled water was added to the mixture, and the absorbance was measured at 540 nm. Lipase activity was determined using the method proposed by Bülow and Mosbach [31], with minor adjustments. The reaction mixture was composed of Tris-HCl buffer, p-nitrophenyl butyrate, and fish intestine homogenate. The hydrolysis rate of p-nitrophenyl butyrate was assessed at a wavelength of 405 nm over a duration of 5 min, with measurements taken at 30 s intervals. The fish intestine homogenates were combined with an ammonium bicarbonate buffer containing azocasein on a shaking apparatus for 19 h. Subsequently, the reaction was halted via the addition of trichloroacetic acid, followed by centrifugation (10,000 ×*g* for 5 min). The ultimate supernatant was mixed with NaOH, and the resultant optical density was recorded at a wavelength of 450 nanometers.

### 2.6. Hematology Parameters

At the end of the experiment, 12 starved fish from each treatment (four fish per tank) were sampled and anesthetized with 0.2 mL/L phenoxyethanol. Blood was then drawn from the caudal vein and collected into heparin-free tubes. These tubes were centrifuged at 1600× *g* for 10 min to separate the serum, which was subsequently stored at −−70°C for further analysis. Erythrocytes (RBC) and leukocytes (WBC) were counted using a haemocytometer. The method of cyanmethemoglobin was employed to measure the concentration of hemoglobin (Hb). The measurement of hematocrit (Hct) percentage was achieved through the use of microhematocrit capillary tubes [32]. Mean corpuscular volume (MCV), mean corpuscular haemoglobin concentration (MCHC), and mean corpuscular haemoglobin (MCH) were measured according to the following formulas [33]:

  $$\begin{array}{c} \text{Mean corpuscular volume}:\text{ MCV }\left(\text{fl}\right)=\left(\frac{\text{Hct}}{\text{RBC}}\right)\times 10, \end{array}$$

  $$\begin{array}{c} \text{Mean corpuscular hemoglobin concentration: MCHC }\left(\%\right)=\left(\frac{\text{Hb}}{\text{Hct}}\right)\times 100, \end{array}$$

  $$\begin{array}{c} \text{Mean corpuscular hemoglobin: MCH }\left(\text{fl}\right)=\left(\frac{\text{Hb}}{\text{RBC}}\right)\times 10. \end{array}$$

### 2.7. Immunity and Antioxidant Biomarkers

Serum samples from 12 individuals per treatment were used to analyze immune and antioxidant factors. Aspartate aminotransferase (AST), alanine transaminase (ALT), alkaline phosphatase (ALP), catalase (CAT), glutathione peroxidase (GPX), malondialdehyde (MDA), superoxide dismutase (SOD), and total proteins (TPs) were assayed using diagnostic reagent kits (Pars Azmon, Iran). The turbidimetric method was applied to detect lysozyme activity (LYS) as reported in Ellis [34]. The method of Yano et al. [35] was employed to measure serum alternative complement (ACH50) activity. Total immunoglobulin content was determined according to the Siwicki protocol [36] using polyethylene glycol precipitation. The level of biochemical parameters is expressed in units per liter, while the levels of antioxidant and immune parameters are expressed in units per milliliter.

### 2.8. Genes Expression

The RNA from the intestine of four fish per tank was extracted using the EZ-10 spin column total RNA mini-prep kit (Takara, Japan). The quantity and quality of the extracted RNA were evaluated using the Nanodrop 2000 spectrophotometer (Thermo Fisher Scientific, Wilmington, USA) and gel electrophoresis, respectively. Subsequently, complementary DNA (cDNA) was synthesized using the RevertAid initial strand cDNA synthesis kit (Takara, Japan). The primers for the selected target and reference genes were designed using Oligo 5 primer analysis software (Table 2). For realtime PCR analysis, the YTA Sybr Green qPCR master mix 2x (Yekta Tajhiz, Iran) was utilized following the manufacturer 's instructions. Amplification was carried out in a realtime PCR detection system with an initial denaturing step of 95°C for 15 min, followed by 40 cycles of 93°C for 15 s, 59°C for 35 s, and 72°C for 20 s, with a melting temperature range of 65°C–95°C for 10 s.

The relative mRNA level was determined using the 2^-ΔΔCT^ method [37], with the GAPDH gene serving as a reference gene for normalizing the expression of the target genes. The control treatment was used as the calibrator in the normalization method.

### 2.9. Data Analysis

The normality of the data and equality of the variances were assessed using the Shapiro–Wilk test and Levene's test, respectively. To examine significant differences between treatments, a one-way analysis of variance (ANOVA) was conducted. Disparities among treatments were further analyzed using the Duncan post hoc test. Statistical significance was set at *p* < 0.05. All the data were subjected to analysis using version 22 of the SPSS software.

## 3. Results

### 3.1. Growth Performance

The results of growth performance after an 8-week feeding trial are presented in Table 3. ILE150 and ILE200 showed significantly (*p* < 0.05) higher values for FW and WG compared to the control group. Notably, a substantial impact was observed in the FCR among the treatments, with ILE200 showing the lowest value (*p* < 0.05). Feeding with an ILE-supplemented diet did not elicit any significant effect on the survival rate (*p* > 0.05).

### 3.2. Digestive Enzymes

The results of digestive enzyme activities in rainbow trout fed with control and ILE-supplemented diets are presented in Table 4. The activity levels of trypsin, pepsin, proteases, and amylase were notably increased at ILE150 and ILE200 compared to the control treatment (*p* < 0.05). Moreover, the activity of lipase at ILE200 was significantly higher than in the other treatments. No significant (*p* > 0.05) difference was found in chymotrypsin activity among treatments (Table 4).

### 3.3. Hematological Indices

Experimental diets significantly affected hematological indices, including RBC, WBC, Htc, Hb, MCV, MCH, and MCHC, with the highest levels observed in ILE150 and ILE200 (*p* < 0.05; Table 5).

### 3.4. Serum Liver Enzymes

**Figure 1:**
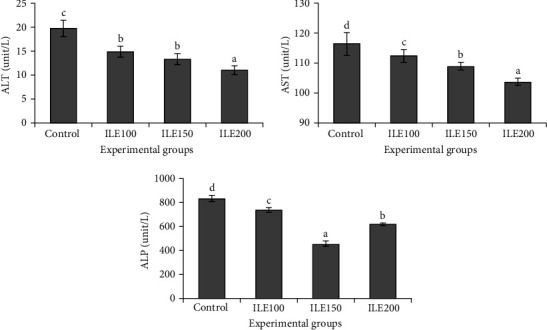
Alanine aminotransferase (ALT), aspartate aminotransferase (AST), and alkaline phosphatase (ALP) in the serum of rainbow trout (*Oncorhynchus mykiss*) fed with 0 (control), 100 (ILE100), 150 (ILE150), and 200 (ILE200) mg of Ivy leaf extract/kg of feed, for 8 weeks. Different letters designate significant differences (*p* < 0.05) as determined by Duncan's post hoc tests (mean ± SD), (*n* = 6).

Significant differences were observed in the serum activities of ALT, AST, and ALP among experimental treatments (Figure 1). The ILE200 group exhibited the lowest levels of ALT and AST. For ALT, both the ILE100 and ILE150 groups had lower values than the control group but higher than those in ILE200, with no significant (*p* > 0.05) differences between ILE100 and ILE150. Notably, a decrease in ALT was observed in a dose-dependent manner, reaching its lowest point in ILE200. The ALP level in ILE150 was significantly lower than in the control and other experimental groups. Additionally, ALP levels in ILE200 were lower than those in ILE100, with both being significantly reduced compared to the control group. An increase in dietary ILE inclusion was associated with a decrease in ALT, AST, and ALP activities (Figure 1, *p* < 0.05).

### 3.5. Antioxidant Factors

**Figure 2:**
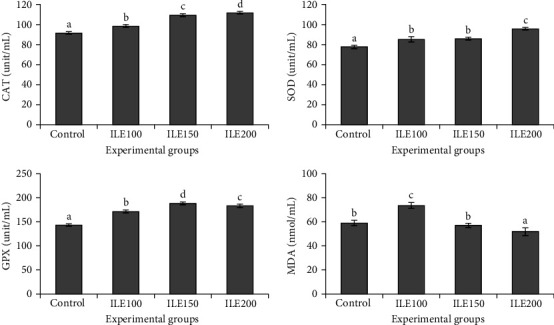
Catalase (CAT), superoxide dismutase (SOD), glutathione peroxidase (GPX), and malondialdehyde (MDA) of rainbow trout (*Oncorhynchus mykiss*) fed with 0 (control), 100 (ILE100), 150 (ILE150), and 200 (ILE200) mg of Ivy leaf extract/kg of feed, for 8 weeks. Different letters designate significant differences (*p* < 0.05) as determined by Duncan's post hoc tests (mean ± SD), (*n* = 6).

Serum antioxidant enzymes, including CAT, SOD, and GPX, exhibited a significant (*p* < 0.05) increase in the experimental groups compared to the control (Figure 2). Additionally, these enzymes demonstrated an ascending trend paralleling the elevation of dietary ILE levels. In contrast, the serum MDA level (Figure 2) significantly increased in ILE100. However, MDA levels decreased significantly (*p* < 0.05) in the ILE150 and ILE200 groups (Figure 2).

### 3.6. Immune Factors

**Figure 3:**
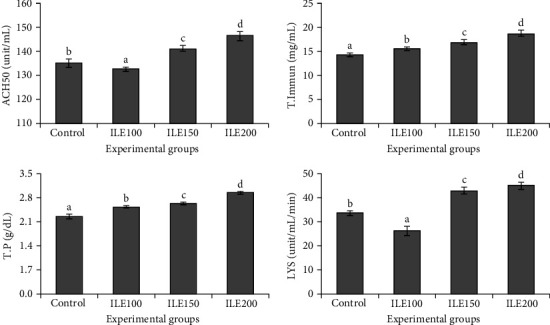
The alternative complement activity (ACH50), total immunoglobulin (T.Immun), total protein (TP) and lysozyme activity (LYS) in the serum of rainbow trout (*Oncorhynchus mykiss*) fed with 0 (control), 100 (ILE100), 150 (ILE150), and 200 (ILE200) mg of Ivy leaf extract/kg of feed, for 8 weeks. Different letters designate significant differences (*p* < 0.05) as determined by Duncan's post hoc tests (mean ± SD), (*n* = 6).

ACH50, total immunoglobulin, TP, and lysozyme levels were significantly (*p* < 0.05) higher in ILE150 and ILE200 compared to the control (Figure 3).

### 3.7. Genes Expression

**Figure 4:**
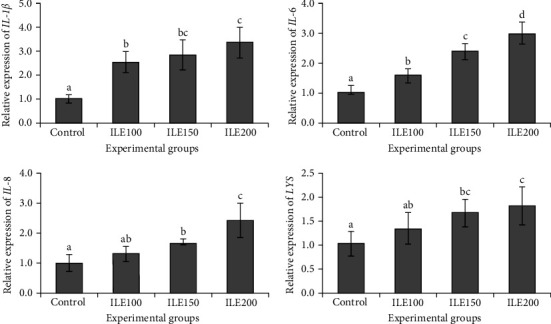
Relative expression of interleukin-1β (IL-1β), interleukin-6 (IL-6), interleukin-8 (IL-8), and lysozyme (LYS) of rainbow trout (*Oncorhynchus mykiss*) fed with 0 (control), 100 (ILE100), 150 (ILE150), and 200 mg (ILE200) of Ivy leaf extract/kg of feed, for 8 weeks. Different letters designate significant differences (*p* < 0.05) as determined by Duncan's post hoc tests (mean ± SD), (*n* = 6).

The results of the relative mRNA expression of immune-related genes showed a remarkable upregulation of *IL-1β* and *IL-6* in all ILE-fed treatments, that is, ILE100, ILE150 and ILE200 compared to the control (Figure 4). Moreover, the transcripts of *IL-8* and *LYS* were significantly upregulated in ILE150 and ILE200 treatments compared to the control (Figure 4, *p* < 0.05).

## 4. Discussion

The results obtained from the present study demonstrated positive effects of ILE-supplemented diets on growth performance, immunity, and antioxidant parameters in rainbow trout. After an 8-week feeding trial with ILE-supplemented diets, it was observed that the experimental diets significantly enhanced fish growth, with a particularly notable increase at dosages of 150 and 200 mg of ILE/kg diet. Dietary supplementation with natural plant extracts rich in polyphenolic compounds has been shown to enhance fish growth and oxidative status. For instance, a study on Senegalese sole (*Solea senegalensis*) revealed that diets supplemented with curcumin and grape seed extracts led to improved growth performance compared to control diets [9]. Similarly, research involving Asian sea bass (*Lates calcarifer*) indicated that dietary polyphenols positively influenced growth performance [38]. Additionally, the beneficial effects of dietary herbal polyphenolic compounds have been reported in various fish species [17, 39–41], highlighting their potential as effective feed additives in aquaculture to promote growth and overall health. The effectiveness of herbal extracts is believed to stem from the presence of biologically active compounds [42]. These bioactive compounds are supposed to contribute to pleiotropic effects. Certain polyphenols, such as curcumin, are shown to influence the expression of genes related to growth factors, including insulin-like growth factor gene (IGFs) [9]. These factors are essential for muscle growth and development. Moreover, herbal polyphenols may enhance digestive enzyme activity, leading to improved nutrient absorption and feed conversion efficiency. This effect contributes to better growth performance as fish can more effectively utilize the nutrients in their diet. So, herbal bioactive compounds, including polyphenols and nonpolyphenolic compounds like saponin, may exert their beneficial effects on growth performance by promoting growth factors and improving digestive efficiency. Considering the prebiotic properties of polyphenols [43] and the growth-promoting effects of other bioactive compounds like saponin [44], another beneficial action of these compounds is their ability to stimulate the growth of beneficial gut microbiota while simultaneously inhibiting the growth of pathogenic bacteria [45–47]. This dual action results in improved intestinal health and enhanced nutrient absorption.

Crucial enzymatic activities, including pepsin, trypsin, amylase, and lipase, play a pivotal role in determining the functioning of the digestive system in fish [48]. In this study, an increase in trypsin, pepsin, proteases, lipase, and amylase activities was observed in fish fed with ILE-supplemented diets. Similar increases in digestive enzyme activities have been reported in other research following the inclusion of different herbal polyphenols in feeds [6, 49–51]. The boosting effects of herbal polyphenols on digestive enzyme activity and nutrition, which result in enhanced growth performance, may be attributed to several factors. These include the role of polyphenols in increasing the expression of genes involved in enzyme synthesis and secretion in the digestive tract [52], their antibacterial properties that help maintain a healthy gut microbiota [53], which further aids in digestion and nutrient absorption and their antioxidant properties that protect the intestinal epithelium, leading to improved digestive health [54]. Additionally, polyphenols can act as appetite stimulants and promote the secretion of mucus in the gut, which serves as a protective barrier and facilitates nutrient transport [54]. Conversely, some studies have shown that when fish are administered polyphenols, such as quercetin [51], p-Coumaric acid [6], and trans-Cinnamic acid [55], the activity of digestive enzymes decreases. The controversial effects of polyphenols on digestive enzyme activity in fish can be attributed to several factors, including the type of polyphenol, the dosage administered, and the specific fish species involved.

Hematological indices are invaluable factors in the evaluation of fish health [56]. In this study, the addition of ILE to fish diets significantly affected hematological indices, increasing all parameters except for the percentages of lymphocytes and monocytes. Numerous studies have demonstrated the stimulating effects of herbal extracts on the hematological indices of various fish species [57–59]. Mişe Yonar [60] showed that dietary ellagic acid, a natural polyphenol, caused an elevation in the RBC count, Hb concentration, and Hct level in rainbow trout. Aligning with these results, Yılmaz and Ergün [61] found that a supplemented diet with varying concentrations of trans-Cinnamic acid revealed a boosting effect on the hematological parameters of rainbow trout, including blood granulocyte percentage. In other studies, dietary administration of tea polyphenols in coho salmon (*Oncorhynchus kisutch*) [62], agricultural by-products polyphenols in convict cichlid (*Amatitlania nigrofasciata*) [63], combined chestnut wood and olive polyphenols in Asian sea bass (*L. calcarifer*) [38] and common carp (*Cyprinus carpio*) [15], and olive leaf polyphenols in common carp [64], all demonstrated similar increasing effects on hematological indices. The inductive effects of polyphenols on blood parameters are likely due to the impact of these bioactive compounds on various aspects of the recipients' physiology. The antioxidant properties of herbal polyphenols [65], along with their potential effects on stimulating eryhtropoiesis [66, 67], enhancing immunity [15, 68], improving nutrition [69, 70], and reducing inflammation in fish [71], can collectively contribute to the observed increases in hematological indices following the inclusion of herbal polyphenols in fish diets.

Alteration in the activities of ALP, ALT, and AST enzymes in fish tissues and plasma can provide valuable insights into the physiological status of fish in response to various environmental factors, toxicants, or dietary changes [56, 72]. Therefore, monitoring their fluctuations is an effective method for assessing fish health and stress levels. Feeding fish with ILE-supplemented diets significantly reduced the plasma levels of ALT, AST, and ALP activities. This reduction was dose-dependent, with the greatest decrease observed at the highest dose of ILE application. A decrease in liver enzyme activities, particularly ALT, AST, and ALP, following the administration of herbal polyphenolic compounds as well as phytosterols in fish feed, suggests that ILE-supplemented diets probably attribute to hepatoprotective, antioxidant, anti-inflammatory, and metabolic roles, ultimately contributing to improved liver health and function as sustained by some researchers. The oral administration of silymarin, a polyphenolic flavonoid derived from milk thistle, was used on rainbow trout, and the observed reduction in ALT, ALP, and AST activities was attributed to the hepatoprotective effects of this herbal bioactive [73]. Ghafarifarsani et al. [74] pointed out that the antioxidant properties of the combined inclusion of *Malvae sylvestris*, *Origanum vulgare*, and *Allium hirtifolium* boiss, which are all herbals rich in polyphenols, can decrease the levels of AST, ALT, and ALP activities in common carp. In another research, largemouth bass juveniles (*Micropterus salmoides*) subjected to hyperglycemia-induced metabolic disorder were fed a mulberry leaf powder-supplemented feed. The results of this survey revealed that the anti-inflammatory effects of this polyphenol-rich herbal supplement effectively alleviated the negative effects of high starch-induced hepatic oxidative stress [75]. Dietary phytosterol supplementation enhanced glucose utilization and metabolism in juvenile largemouth bass (*M. salmoides*) fed a high-starch diet, thereby reducing the negative effects of glycemic stress [76]. These beneficial effects may be attributed to increased activities of protease and lipase, which in turn contributed to the recovery of feed utilization efficiency under high-starch stress. The reduction in liver enzyme activity following the administration of ILE can be attributed to multiple mechanisms. Altogether, these mechanisms underscore the potential therapeutic benefits of ILE in supporting and maintaining liver health.

The assessment of SOD, CAT, and GPX activity, recognized as major antioxidant enzymes, serves as an indicator of antioxidant capacity in aquatic organisms [77]. Changes in the activity of SOD, CAT, and GPX indicate the impact of different parameters, including environmental stressors and feeding regimes. By measuring the activities of these enzymes, an insight into the overall health and oxidative stress levels of aquatic organisms can be gained. The results obtained from the present experiment suggest that the ILE-supplemented diet significantly enhanced the antioxidant capacity in rainbow trout, evidenced by increased SOD, CAT, and GPX activities, coupled with a decrease in MDA concentration. Similar enhancement in SOD, CAT, and GPX activities, along with a reduction in MDA concentration, has been reported in studies involving herbal extracts on rainbow trout [16, 78–80]. Stimulating effects of a wide range of herbal extracts, which all contain polyphenols, have been shown on other fish species, such as common carp [81, 82], hybrid grouper (*Epinephelus fuscoguttatus* ♀ × *E. lanceolatus* ♂) [83], largemouth bass (*M. salmoides*) [84], striped catfish (*Pangasianodon hypophthalmus*) [85], grass carp (*Ctenopharyngodon Idella*) [86], Siberian sturgeon (*Acipenser baerii*) [87], and black rockfish (*Sebastes schlegelii*) [88]. It is conceivable that suppressing oxidative stress and improving the activity of antioxidant-relevant enzymes in various fish species is closely related to the polyphenols applied.

Enhancing effects of herbal polyphenolic compounds on fish antioxidant enzyme activities may be attributed to several mechanisms. The free radical scavenging potential of polyphenols, flavonoids, and caffeoylquinic acids can neutralize free radicals directly and alleviate oxidative stress in cells [89–94]. Enzyme activity modulation [95], anti-inflammatory effects [96], improving carbohydrate and lipid metabolism [97, 98], and the upregulation of genes encoding antioxidant enzymes [99] are all related to the stimulatory effects of herbal polyphenols on fish antioxidant performance.

The assessment of resistance against invaders can be accomplished by detecting the primary responses of fish immunity in the plasma [100]. The use of herbal supplements as a dietary additive has been confirmed for their ability to modulate the immune system, resulting in the induction of both mucosal and humoral immune responses in aquatic organisms [101–103]. Lysozyme, secreted by leukocytes, plays a vital role in the nonspecific immunity in fish [104]. TP also serves as an indicator of enzymatic activity and protein derivatives involved in immunity [100, 105]. In this study, fish fed with ILE-supplemented diets demonstrated a significant increase in blood immune-related parameters, including TP, total immunoglobulin, and lysozyme. Consistent with the present study, dietary *Aloysia citrodora* enhanced total immunoglobulin and lysozyme in rainbow trout [78, 106]. Trans-Cinnamic acid-supplemented diets have also been shown to elevate immune parameters in rainbow trout, including total immunoglobulin, lysozyme, and the phagocytic index [61]. Additionally, various herbal extracts, including *Stachys lavandulifolia* Vahl extract [107], *Anethum graveolens* [108], *Malvae sylvestris* [109], *Allium hirtifolium* [110], and *Silybum marianum* [111], have demonstrated immune-enhancing effects in rainbow trout. The augmented immune response observed in the current investigation, similar to the effects seen with other plant-derived polyphenols like silymarin [112], curcumin [113], and tea polyphenols [114], may be attributed to the enhancement of local intestinal immunity, a direct consequence of the administration of ILE. These bioactive compounds can influence various immune-related pathways, contributing to improve local immunity in the fish's gut. Among these biological pathways is the enhancement of the humoral components, such as immunoglobulins and lysozyme, which are crucial for both innate and adaptive immunity [115]. Enhancing ACH50 activity may be achieved as another pathway to improve the complement system [116].

Additionally, the expressions of cytokine and immune-related genes (*IL-1β*, *IL-6*, *IL-8*, and *LYS*) support the findings that the ILE-supplemented diet enhances fish immune responses. Cytokines are essential for the immune system's proper functioning and function as signaling molecules in the immune system, playing a crucial role in cell-to-cell communication. Applying ILE-supplemented diets significantly upregulated the expression of intestinal *IL-1β*, *IL-6*, *IL-8*, and *LYS* genes in a dose-dependent manner. In alignment with the findings of the present study, Nootash et al. [117] showed an upregulation of spleen *IL-1β* and *IL-6*, liver *IL-6* and *IL-8*, and kidney *IL-8* genes after green tea administration in rainbow trout. In another study, the application of lower doses of dietary *Urtica dioica* significantly increased the expression of *IL-1β*, *IL-6*, and *IL-8* genes in rainbow trout [118]. Other research also reported increasing effects of different herbal bioactive compounds like trans-Cinnamic acid in rainbow trout [61], olive leaf extract in rainbow trout [119], and *Glycyrrhiza uralensis* extract in yellow catfish (*Pelteobagrus fulvidraco*) [120]. The upregulation of cytokine genes and lysozyme in fish after the dietary application of ILE can be attributed to several factors. Cytokines like *IL-1β*, *IL-6*, and *IL-8* are pro-inflammatory cytokines that play crucial roles in the immune response of fish [10, 121]. The applied bioactive compounds are believed to have the potential to stimulate the immune system, leading to an increased production of these cytokines as part of the experimental fish's defense mechanism against invaders [122]. Additionally, these herbal bioactive substances can modulate immune responses by influencing the expression of immune-related genes [123]. Another possible effect of these herbal compounds is their impact on the signaling pathways that regulate cytokine production [124]. Furthermore, the augmentation of lysozyme gene expression can be part of the enhanced immune response facilitated by polyphenols [15]. However, some other studies revealed a downregulation of cytokines following the application of herbal extract. Magrone et al. [125] demonstrated a decrease in intestinal proinflammatory cytokines (*IL-1β* and *IL-6*) in farmed sea bass after polyphenol administration. Additionally, the administration of tea polyphenols in Koi carp feed resulted in a significant reduction in plasma *IL-1β*, *IL-6*, and lysozyme [114]. In another research, flavonoid-rich extract of orange juice resulted in a notable downregulation of inflammatory genes, including *IL-1β*, *IL-6*, and *TNFα* [126]. These contradictory results can be attributed to several factors, such as differences in the type and concentration of the applied polyphenols, cellular context and environment, signaling pathway, and experimental conditions.

## 5. Conclusion

In conclusion, the results demonstrate that ILE-supplemented diets, especially at 150–200 mg inclusion, significantly enhance growth performance, digestive enzyme activity, hematological indices, antioxidant capacity, and immune responses in rainbow trout. These findings suggest that ILE supplementation can be a valuable strategy to improve fish health and productivity in aquaculture. However, future research should explore the long-term effects of ILE supplementation on fish health and performance, including potential impacts on disease resistance. Understanding the molecular mechanisms underlying the observed physiological changes would also provide valuable insights into the role of ILE in fish nutrition and immune modulation.

## Figures and Tables

**Table 1:** Ingredients and composition of basal diet.

Ingredients	(g/kg dry matter)
Soybean meal^a^	200
Fish meal^b^	310
Wheat gluten^c^	100
Wheat meal	166
Poultry by-product^d^	130
Fish oil	40
Soybean oil	30
Methionine^e^	4
Phytase^f^	8
Lysine^e^	7
Vitamins premix^g^	2.5
Minerals premix^g^	2.5
Chemical composition^h^	
Crude protein	425
Crude fat	163
Crude ash	79.5
Crude fibre	30.8
Moisture	88.2
Gross energy (kJ/*g*)	19.4

*Note:* Feed formulation was prepared according to Yousefi et al. [25].

^a^Soyabean Co., Gorgan, Iran (crude protein 45.2%).

^b^Peygir Co., Gorgan, Iran (crude protein 58.8%).

^c^Shahdineh Aran Co., Isfahan, Iran (crude protein 78.3%).

^d^Peygir Co., Gorgan, Iran (crude protein 51.0%).

^e^Golbid Co., Tehran, Iran (10,000 IU).

^f^CheilJedang Co., Seul, Korea.

^g^The premix provided following amounts per kg of feed: A: 1000 IU; D3: 5000 IU; E: 20 mg; B5: 100 mg; B2: 20 mg; B6: 20 mg; B1: 20 mg; H: 1 mg; B9: 6 mg; B12: 1 mg; B4: 600 mg; C: 50 mg; Mg: 350 mg; Fe: 13 mg; Co: 2.5 mg; Cu: 3 mg; Zn: 60 mg; Se: 0.3 mg; I: 1.5 mg; Mn: 10 mg.

^h^Determined according to AOAC [26].

**Table 2:** The list of primers for amplifying genes.

Gene	Primer	T (°C)	Product length (pb)	NCBI reference	Primer efficiency
*IL-1β*	F: 5′ TATCCCATCACCCCATCAC 3′	57.72	170	XM_036979104.1	92.6
	R: 5′ CGACTCCAACTCCAACACT 3′	57.01			

*IL-6*	F: 5′ TGCTCGTGGTGATGGTAGTT 3′	59.03	176	XM-021572949.2	93.7
	R: 5′ TGTTGGTCTCGGAAGTTGAT 3′	56.78			

*IL-8*	F: 5′ GGCAGATTCAAACTCTCCAC 3′	56.42	128	NM_01124362.1	92.3
	R: 5′ TACGGTTGTTATCTAGCTGGT 3′	57.19			

*Lys*	F: 5′ GTTCTCCTGCTTGTGGCTG 3′	58.75	128	NM_001124716.1	94.1
	R: 5′ TTTGACAGGCAAACCCAGTT 3′	58.14			

*GAPDH*	F: 5′ TCGTAAGACAGGATTGAGGC 3′	57.04	148	NM-001124209.1	95.3
	R: 5′ GGCAACAATCTCAACTCCCT 3′	57.79			

Abbreviations: *GAPDH*, glyceraldehyde-3-phosphate dehydrogenase; *IL-1β*, interleukin 1β; *IL-6*, interleukin-6; *IL-8*, interleukin-8; *Lys*, lysozyme.

**Table 3:** Growth efficiency of rainbow trout (*Oncorhynchus mykiss*) fed with 0 (control), 100 (ILE100), 150 (ILE150), and 200 (ILE200) mg of Ivy leaf extract/Kg of feed, for 8 weeks.

	IW (g)	FW (g)	WG (g)	FCR	SGR (%/day)	FI	SR (%)
Control	6.34 ± 0.36^a^	31.25 ± 0.15^a^	24.91 ± 0.42^a^	1.20 ± 0.02^b^	2.85 ± 0.10^ab^	30	100
ILE100	6.56 ± 0.37^a^	31.56 ± 0.18^a^	25.00 ± 0.40^a^	1.24 ± 0.02^c^	2.80 ± 0.10^a^	31	100
ILE150	6.13 ± 0.14^a^	32.41 ± 0.23^b^	26.27 ± 0.27^b^	1.21 ± 0.01^bc^	2.97 ± 0.04^b^	32	100
ILE200	6.50 ± 0.36^a^	33.06 ± 0.50^c^	26.55 ± 0.62^b^	1.13 ± 0.02^a^	2.90 ± 0.10^ab^	30	100
*p*-value	0.134	<0.001	<0.001	<0.001	0.033	—	—

*Note:* Different letters designate significant differences (*p* < 0.05) as determined by Duncan's post hoc tests (mean ± SD), (*n* = 6).

Abbreviations: FCR, feed conversion rate; FI, feed intake; FW, final weight; IW, initial weight; SGR, specific growth rate; SR, survival rate; WG, weight gain.

**Table 4:** Digestive enzymes of rainbow trout (*Oncorhynchus mykiss*) fed with 0 (control), 100 (ILE100), 150 (ILE150), and 200 (ILE200) mg of Ivy leaf extract/Kg of feed, for 8 weeks.

	Trypsin (*µ*mol /mg p)	Chymotrypsin (µmol/mg p)	Pepsin (µmol/mg p)	Proteases (u/mg p)	Amylase (u/mg p)	Lipase (u/mg p)
Control	0.047 ± 0.003^a^	0.016 ± 0.005^a^	0.021 ± 0.001^a^	2.607 ± 0.165^a^	16.263 ± 0.400^a^	1.102 ± 0.067^a^
ILE100	0.051 ± 0.002^b^	0.013 ± 0.001^a^	0.021 ± 0.001^a^	2.683 ± 0.096^a^	15.317 ± 0.620^a^	1.112 ± 0.077^a^
ILE150	0.059 ± 0.004^c^	0.015 ± 0.001^a^	0.025 ± 0.002^b^	3.260 ± 0.437^b^	18.220 ± 0.617^b^	1.163 ± 0.090^a^
ILE200	0.061 ± 0.002^c^	0.015 ± 0.000^a^	0.028 ± 0.002^c^	3.310 ± 0.161^b^	22.640 ± 1.59^c^	1.270 ± 0.084^b^
*p*-Value	<0.001	0.475	<0.001	<0.001	<0.001	0.006

*Note:* Different letters designate significant differences(*p* < 0.05)as determined by Duncan post hoc tests (mean ± SD), (*n* = 6).

**Table 5:** Hematological indices of rainbow trout (*Oncorhynchus mykiss*) fed with 0 (control), 100 (ILE100), 150 (ILE150), and 200 (ILE200) mg of Ivy leaf extract/Kg of feed, for 8 weeks.

	RBC (10^6^/mL)	WBC (10^3^/mL)	Htc (%)	Hb (g/dL)	MCV (fl)	MCH (fl)	MCHC (%)	Neutr (%)	Lymph (%)	Monocy (%)
Control	1.28 ± 0.05^a^	3.13 ± 0.36^a^	36.33 ± 1.21^a^	6.80 ± 0.14^a^	285.94 ± 11.84^a^	53.51 ± 1.55^a^	18.72 ± 0.30^b^	12.16 ± 1.47^a^	83.33 ± 3.27^b^	3.83 ± 1.47^a^
ILE100	1.35 ± 0.03^b^	3.45 ± 0.19^a^	40.67 ± 2.16^b^	7.41 ± 0.27^b^	301.98 ± 14.90^b^	55.06 ± 0.99^b^	18.26 ± 0.80^ab^	13.00 ± 0.89^a^	82.83 ± 1.17^b^	3.50 ± 1.05^a^
ILE150	1.60 ± 0.02^c^	4.80 ± 0.46^b^	50.00 ± 1.41^c^	9.10 ± 0.14^c^	312.15 ± 7.13^b^	56.82 ± 0.78^c^	18.21 ± 0.41^ab^	15.33 ± 1.21^b^	80.67 ± 1.97^ab^	3.33 ± 1.21^a^
ILE200	1.73 ± 0.03^d^	4.57 ± 0.08^b^	54.00 ± 1.41^d^	9.55 ± 0.10^d^	312.80 ± 9.20^b^	55.32 ± 0.93^b^	17.70 ± 0.61^a^	16.33 ± 1.21^b^	78.33 ± 2.42^a^	4.67 ± 1.63^a^
*p*-Value	<0.001	<0.001	<0.001	<0.001	0.001	0.001	0.041	<0.001	0.005	0.357

*Note:* Different letters designate significant differences(*p* < 0.05)as determined by Duncan post hoc tests (mean ± SD), (*n* = 6).

Abbreviations: Hb, hemoglobin; Htc, hematocrit; Lymph, lymphocytes; MCH, mean corpuscular hemoglobin; MCHC, mean corpuscular hemoglobin content; MCV, mean corpuscular volume; Monocy, monocytes; Neutr, neutrophils; RBC, red blood cell; WBC, white blood cell.

## Data Availability

The data that support the findings of this study are available from the corresponding author upon reasonable request.

## References

[1] Etxeberria U., Fernández-Quintela A., Milagro F. I., Aguirre L., Martínez J. A., Portillo M. P. (2013). Impact of Polyphenols and Polyphenol-Rich Dietary Sources on Gut Microbiota Composition. *Journal of Agricultural and Food Chemistry*.

[2] Ahmadifar E., Yousefi M., Karimi M. (2021). Benefits of Dietary Polyphenols and Polyphenol-Rich Additives to Aquatic Animal Health: An Overview. *Reviews in Fisheries Science and Aquaculture*.

[3] Tungmunnithum D., Thongboonyou A., Pholboon A., Yangsabai A. (2018). Flavonoids and Other Phenolic Compounds from Medicinal Plants for Pharmaceutical and Medical Aspects: An Overview. *Medicines*.

[4] Faheem M., Abbas R. Z., Liaqat I., Hoseinifar S. H., Maneepitaksanti W., Doan H. V. (2022). Bio-Active Components in Medicinal Plants: A Mechanistic Review of Their Effects on Fish Growth and Physiological Parameters – A Review. *Annals of Animal Science*.

[5] Vendrame S., Klimis-Zacas D. (2015). Anti-Inflammatory Effect of Anthocyanins via Modulation of Nuclear Factor-*κ*B and Mitogen-Activated Protein Kinase Signaling Cascades. *Nutrition Reviews*.

[6] Ahmadifar E., Kalhor N., Dawood M. A. O., Ahmadifar M., Moghadam M. Shahriari, Yousefi M. (2021). Effects of Dietary *p*-Coumaric Acid on the Growth Performance, Digestive Enzyme Activity, Humoral Immunity and Immune-Related Gene Expression in Common Carp, *Cyprinus carpio*. *Aquaculture Nutrition*.

[7] Paolucci M., Volpe M. G., Costantini S. (2016). Polyphenol Enriched Diets for Aquaculture Applications. *Journal of International Society of Antioxidants in Nutrition and Health*.

[8] Guo H., Lin W., Hou J. (2018). The Protective Roles of Dietary Selenium Yeast and Tea Polyphenols on Growth Performance and Ammonia Tolerance of Juvenile Wuchang Bream (*Megalobrama amblycephala*). *Frontiers in Physiology*.

[9] Xavier M. J., Conceição L. E. C., Valente L. M. P. (2021). Dietary Natural Plant Extracts Can Promote Growth and Modulate Oxidative Status of Senegalese Sole Postlarvae under Standard/Challenge Conditions. *Animals (Basel)*.

[10] Shohreh P., Mohammadzadeh S., Mahboub H. H. (2024). Growth Performance, Hematological Profile, and Related Genes Expression in Goldfish (*Carassius auratus*) Fed on Rosmarinic Acid-Enriched Diets and Subjected to Ambient Ammonia. *Aquaculture*.

[11] Jiang L., Zhou X., Yu J. (2022). Fermented Tea Residue Improved Growth Performance, Liver Antioxidant Capacity, Intestinal Morphology and Resistance to *Aeromonas hydrophila* Infection in Juvenile Largemouth Bass (*Micropterus salmoides*). *Frontiers in Marine Science*.

[12] Santana P. A., Jara-Gutiérrez C., Mellado M. (2021). Effects of Elderflower Extract Enriched with Polyphenols on Antioxidant Defense of Salmon Leukocytes. *Electronic Journal of Biotechnology*.

[13] Fabrikov D., Varga Á. T., García M. C. V. (2024). Antimicrobial and Antioxidant Activity of Encapsulated Tea Polyphenols in Chitosan/Alginate-Coated Zein Nanoparticles: A Possible Supplement Against Fish Pathogens in Aquaculture. *Environmental Science and Pollution Research*.

[14] Shahidi F., Ambigaipalan P. (2015). Phenolics and Polyphenolics in Foods, Beverages and Spices: Antioxidant Activity and Health Effects – A Review. *Journal of Functional Foods*.

[15] Jahazi M. A., Hoseinifar S. H., Jafari V., Hajimoradloo A., Van Doan H., Paolucci M. (2020). Dietary Supplementation of Polyphenols Positively Affects the Innate Immune Response, Oxidative Status, and Growth Performance of Common Carp, *Cyprinus carpio*, L. *Aquaculture*.

[16] Hoseinifar S. H., Shakouri M., Yousefi S. (2020). Humoral and Skin Mucosal Immune Parameters, Intestinal Immune Related Genes Expression and Antioxidant Defense in Rainbow Trout (*Oncorhynchus mykiss*) Fed Olive (*Olea Europea* L.) Waste. *Fish and Shellfish Immunology*.

[17] Safari R., Hoseinifar S. H., Imanpour M. R., Mazandarani M., Sanchouli H., Paolucci M. (2020). Effects of Dietary Polyphenols on Mucosal and Humoral Immune Responses, Antioxidant Defense and Growth Gene Expression in Beluga Sturgeon (*Huso huso*). *Aquaculture*.

[18] FAO (2024). The State of World Fisheries and Aquaculture 2024 – Blue Transformation in Action.

[19] Bezruk I., Marksa M., Georgiyants V., Ivanauskas L., Raudone L. (2020). Phytogeographical Profiling of Ivy Leaf (*Hedera helix* L.). *Industrial Crops and Products*.

[20] Shokry A., El-Shiekh R., Kamel G., Ramadan A. (2022). Phytochemical Contents, Biological Activities and Therapeutic Applications of *Hedera helix* (Ivy Leaf) Extracts: A Review. *The Natural Products Journal*.

[21] Lutsenko Y., Bylka W., Matlawska I., Darmohray R. (2010). *Hedera helix* as a Medicinal Plant. *Herba Polonica*.

[22] Pop C. E., Parvu M., Arsene A. L. (2017). Investigation of Antioxidant and Antimicrobial Potential of Some Extracts From *Hedera helix* L. *Farmacia*.

[23] Miser-Salihoglu E., Akaydin G., Caliskan-Can E., Yardim-Akaydin S. (2013). Evalution of Antioxidant Activity of Various Herbal Folk Medicines. *Journal of Nutrition and Food Sciences*.

[24] Parvu M., Vlase L., Parvu A. E., Rosca-Casian O., Gheldiu A.-M., Parvu O. (2015). Phenolic Compounds and Antifungal Activity of, *Hedera helix*, L. (Ivy) Flowers and Fruits. *Notulae Botanicae Horti Agrobotanici Cluj-Napoca*.

[25] Yousefi M., Vatnikov Y. A., Kulikov E. V. (2021). Effects of Dietary *Hibiscus sabdariffa* Supplementation on Biochemical Responses and Inflammatory-Related Genes Expression of Rainbow Trout, *Oncorhynchus mykiss*, to Ammonia Toxicity. *Aquaculture*.

[26] Association of Officiating Analytical Chemists (2005). *AOAC, Official Method of Analysis*, Method 935.14 and 992.24..

[27] Giannenas I., Triantafillou E., Stavrakakis S. (2012). Assessment of Dietary Supplementation with Carvacrol or Thymol Containing Feed Additives on Performance, Intestinal Microbiota and Antioxidant Status of Rainbow Trout (*Oncorhynchus mykiss*). *Aquaculture*.

[28] Javahery S., Noori A., Hoseinifar S. H. (2019). Growth Performance, Immune Response, and Digestive Enzyme Activity in Pacific White Shrimp, *Penaeus Vannamei*, Fed Dietary Microbial Lysozyme. *Fish and Shellfish Immunology*.

[29] Bradford M. M. (1976). A Rapid and Sensitive Method for the Quantitation of Microgram Quantities of Protein Utilizing the Principle of Protein-Dye Binding. *Analytical Biochemistry*.

[30] Bernfeld P. (1955). *Amylases*, *α and β*. *Methods in Enzymology*.

[31] Bülow L., Mosbach K. (1987). The Expression in *E. coli* of a Polymeric Gene Coding for an Esterase Mimic Catalyzing the Hydrolysis of p-Nitrophenyl Esters. *FEBS Letters*.

[32] Blaxhall P., Daisley K. (1973). Routine Haematological Methods for use With Fish Blood. *Journal of Fish Biology*.

[33] Afshinfar A., Noori A., Akbarzadeh A., Neitali B. Kalvani, Kamrani E., Sajjadi M. M. (2013). Comparative Study of Some Blood Respiratory Factors Between Mudskipper, *Scartelaos tenuis* and Mullet, *Liza klunzingeri* in the Persian Gulf, Hormozgan Province. (Abstract in English). *Journal of Aquatic Ecology*.

[34] Ellis A. E., Stolen J. S. (1990). Lysozyme Assays. *Techniques in Fish Immunology: Immunological and Pathological Techniques of Aquatic Invertebrates*.

[35] Yano T., Hatayama Y., Matsuyama H., Nakao M. (1988). Titration of the Alternative Complement Pathway Activity of Representative Cultured Fishes. *Nippon Suisan Gakkaishi*.

[36] Siwicki A. K. (1993). Nonspecific Defence Mechanisms Assay in Fish. II. Potential Killing Activity of Neutrophils and Macrophages, Lysozyme Activity in Serum and Organs and Total Immunoglobulin (Ig) Level in Serum. *Fish Diseases Diagnosis and Preventions Methods*.

[37] Livak K. J., Schmittgen T. D. (2001). Analysis of Relative Gene Expression Data Using Real-Time Quantitative PCR and the 2^− ΔΔCT^ Method. *Methods*.

[38] Ahmadi A., Bagheri D., Hoseinifar S. H., Morshedi V., Paolucci M. (2022). Beneficial Role of Polyphenols as Feed Additives on Growth Performances, Immune Response and Antioxidant Status of *Lates calcarifer* (Bloch, 1790) Juveniles. *Aquaculture*.

[39] Van Doan H., Hoseinifar S. H., Hung T. Q. (2020). Dietary Inclusion of Chestnut (*Castanea sativa*) Polyphenols to Nile Tilapia Reared in Biofloc Technology: Impacts on Growth, Immunity, and Disease Resistance Against *Streptococcus agalactiae*. *Fish and Shellfish Immunology*.

[40] Motamedi-Tehrani J., Ebrahimi-Dorcheh E., Goli S. A. H. (2016). Effect of Pistachio (*Pistacia vera*) Hull Extract on Growth Performance, Body Composition, Total Phenolic Compound and Fillets Peroxide Value of Common Carp, *Cyprinus carpio*. *Aquaculture Nutrition*.

[41] Zhong L., Hu Y., Hu Y. (2019). Effects of Dietary Tea Polyphenols on Growth, Immunity and Lipid Metabolism of Juvenile Black Carp *Mylopharyngodon piceus*. *Aquaculture Research*.

[42] Rubió L., Motilva M.-J., Romero M.-P. (2013). Recent Advances in Biologically Active Compounds in Herbs and Spices: A Review of the Most Effective Antioxidant and Anti-Inflammatory Active Principles. *Critical Reviews in Food Science and Nutrition*.

[43] Sateriale D., Facchiano S., Kaldre K. (2023). Benefits of Polyphenol-Based Synbiotics in Crustacean Diet. *Fishes*.

[44] Francis G., Kerem Z., Makkar H. P. S., Becker K. (2002). The Biological Action of Saponins in Animal Systems: A Review. *British Journal of Nutrition*.

[45] Gil-Cardoso K., Ginés I., Pinent M., Ardévol A., Blay M., Terra X. (2016). Effects of Flavonoids on Intestinal Inflammation, Barrier Integrity and Changes in Gut Microbiota during Diet-Induced Obesity. *Nutrition Research Reviews*.

[46] Milutinović M., Dimitrijević-Branković S., Rajilić-Stojanović M. (2021). Plant Extracts Rich in Polyphenols as Potent Modulators in the Growth of Probiotic and Pathogenic Intestinal Microorganisms. *Frontiers in Nutrition*.

[47] Orso G., Solovyev M. M., Facchiano S. (2021). Chestnut Shell Tannins: Effects on Intestinal Inflammation and Dysbiosis in Zebrafish. *Animals*.

[48] Furné M., Morales A. E., Trenzado C. E. (2012). The Metabolic Effects of Prolonged Starvation and Refeeding in Sturgeon and Rainbow Trout. *Journal of Comparative Physiology B*.

[49] Hu H., Liu J., Li Y. (2014). Effects of Dietary Daidzein on Growth Performance, Activities of Digestive Enzymes, Anti-Oxidative Ability and Intestinal Morphology in Juvenile Turbot (*Scophthalmus maximus* L.). *Journal of Fisheries of China*.

[50] Xu G., Xing W., Li T. (2021). Dietary Grape Seed Proanthocyanidins Improved Growth, Immunity, Antioxidant, Digestive Enzymes Activities, and Intestinal Microbiota of Juvenile Hybrid Sturgeon (*Acipenser Baeri* Brandt ♀ × *A. schrenckii* Brandt ♂). *Aquaculture Nutrition*.

[51] Zhai S.-W., Liu S.-L. (2014). Effects of Dietary Quercetin on the Growth Performance, Digestive Enzymes and Antioxidant Potential in the Hepatopancreas of Tilapia (*Oreochromis niloticus*).

[52] Huang W., Yao C., Liu Y. (2022). Effects of Dietary *Eucommia ulmoides* Leaf Extract on Growth Performance, Expression of Feeding-Related Genes, Activities of Digestive Enzymes, Antioxidant Capacity, Immunity and Cytokines Expression of Large Yellow Croaker (*Larimichthys crocea*) Larvae. *British Journal of Nutrition*.

[53] Rodríguez-Daza M. C., Pulido-Mateos E. C., Lupien-Meilleur J., Guyonnet D., Desjardins Y., Roy D. (2021). Polyphenol-Mediated Gut Microbiota Modulation: Toward Prebiotics and Further. *Frontiers in Nutrition*.

[54] Huang T., Che Q., Chen X. (2022). Apple Polyphenols Improve Intestinal Antioxidant Capacity and Barrier Function by Activating the Nrf2/Keap1 Signaling Pathway in a Pig Model. *Journal of Agricultural and Food Chemistry*.

[55] Yılmaz S., Ergun S., Çelik E. Şanver, Yigit M., Bayizit C. (2019). Dietary Trans-Cinnamic Acid Application for Rainbow Trout (*Oncorhynchus mykiss*): II. Effect on Antioxidant Status, Digestive Enzyme, Blood Biochemistry and Liver Antioxidant Gene Expression Responses. *Aquaculture Nutrition*.

[56] Chen H., Luo D. (2023). Application of Haematology Parameters for Health Management in Fish Farms. *Reviews in Aquaculture*.

[57] Harikrishnan R., Balasundaram C., Heo M.-S. (2010). Herbal Supplementation Diets on Hematology and Innate Immunity in Goldfish Against *Aeromonas hydrophila*. *Fish and Shellfish Immunology*.

[58] Anjusha K., Mamun M., Dharmakar P., Shamima N. (2019). Effect of Medicinal Herbs on Hematology of Fishes. *International Journal of Current Microbiology and Applied Sciences*.

[59] Syawal H., Kurniawan R., Effendi I., Austin B. (2021). Fermented Medicinal Herbs Improve Hematological and Physiological Profile of Striped Catfish (*Pangasianodon hypophthalmus*). *F1000Research*.

[60] Yonar S. M. (2019). Growth Performance, Haematological Changes, Immune Response, Antioxidant Activity and Disease Resistance in Rainbow Trout (*Oncorhynchus mykiss*) Fed Diet Supplemented With Ellagic Acid. *Fish and Shellfish Immunology*.

[61] Yılmaz S., Ergün S. (2018). *Trans*-Cinnamic Acid Application for Rainbow Trout (*Oncorhynchus mykiss*): I. Effects on Haematological, Serum Biochemical, Non-Specific Immune and Head Kidney Gene Expression Responses. *Fish and Shellfish Immunology*.

[62] Yu H., Sattanathan G., Yu L., Li L., Xiao Y. (2024). Impact of Nutritional Tea Polyphenols on Growth, Feed Efficiency, Biochemical Traits, Antioxidant Capacity, Haematological Parameters and Immunity in Coho Salmon (*Oncorhynchus kisutch*). *Animals*.

[63] Hoseinifar S. H., Jahazi M. A., Nikdehghan N., Van Doan H., Volpe M. G., Paolucci M. (2020). Effects of Dietary Polyphenols From Agricultural by-Products on Mucosal and Humoral Immune and Antioxidant Responses of Convict Cichlid (*Amatitlania nigrofasciata*). *Aquaculture*.

[64] Zemheri-Navruz F., Acar Ü., Yılmaz S. (2019). Dietary Supplementation of Olive Leaf Extract Increases Haematological, Serum Biochemical Parameters and Immune Related Genes Expression Level in Common Carp (*Cyprinus carpio*) Juveniles. *Fish and Shellfish Immunology*.

[65] Tahir R., Samra F. Afzal, Liang J., Yang S. (2024). Novel Protective Aspects of Dietary Polyphenols Against Pesticidal Toxicity and Its Prospective Application in Rice-Fish Mode: A Review. *Fish and Shellfish Immunology*.

[66] Kannan B., Felix N., Panigrahi A., Ahilan B. (2022). Herbal Extracts Modulate Growth, Immune Responses and Resistance to *Aeromonas hydrophila* Infection in GIFT Tilapia (*Oreochromis niloticus*). *Aquaculture Research*.

[67] Gabriel N. N. (2019). Review on the Progress in the Role of Herbal Extracts in Tilapia Culture. *Cogent Food and Agriculture*.

[68] Pan S., Yan X., Li T. (2022). Impacts of Tea Polyphenols on Growth, Antioxidant Capacity and Immunity in Juvenile Hybrid Grouper (*Epinephelus fuscoguttatus* ♀ × *E. lanceolatus* ♂) Fed High-Lipid Diets. *Fish and Shellfish Immunology*.

[69] Ji R., Li Y., Li X. (2018). Effects of Dietary Tea Polyphenols on Growth, Biochemical and Antioxidant Responses, Fatty Acid Composition and Expression of Lipid Metabolism Related Genes of Large Yellow Croaker (*Larimichthys crocea*). *Aquaculture Research*.

[70] Ji S.-C., Jeong G.-S., Gwang-Soon I. M., Lee S.-W., Yoo J.-H., Takii K. (2007). Dietary Medicinal Herbs Improve Growth Performance, Fatty Acid Utilization, and Stress Recovery of Japanese Flounder. *Fisheries Science*.

[71] Imperatore R., Orso G., Facchiano S. (2023). Anti-Inflammatory and Immunostimulant Effect of Different Timing-Related Administration of Dietary Polyphenols on Intestinal Inflammation in Zebrafish, *Danio rerio*. *Aquaculture*.

[72] Peres H., Santos S., Oliva-Teles A. (2014). Blood Chemistry Profile as Indicator of Nutritional Status in European Seabass (*Dicentrarchus labrax*). *Fish Physiology and Biochemistry*.

[73] Banaee M., Sureda A., Mirvaghefi A. R., Rafei G. R. (2011). Effects of Long-Term Silymarin Oral Supplementation on the Blood Biochemical Profile of Rainbow Trout (*Oncorhynchus mykiss*). *Fish Physiology and Biochemistry*.

[74] Ghafarifarsani H., Hoseinifar S. H., Adorian T. J., Ferrigolo F. R. G., Raissy M., Van Doan H. (2021). The Effects of Combined Inclusion of *Malvae Sylvestris*, *Origanum vulgare*, and *Allium Hirtifolium* Boiss for Common Carp (*Cyprinus carpio*) Diet: Growth Performance, Antioxidant Defense, and Immunological Parameters. *Fish and Shellfish Immunology*.

[75] Xv Z.-C., He G.-L., Wang X.-l., Shun H., Chen Y.-J., Lin S.-M. (2021). Mulberry Leaf Powder Ameliorate High Starch-Induced Hepatic Oxidative Stress and Inflammation in Fish Model. *Animal Feed Science and Technology*.

[76] Liang Q., Huang Y., Zhu N. (2024). Phytosterol Supplementation Enhances the Growth Performance, Feed Utilization, Antioxidant Status and Glucose Metabolism of Juvenile Largemouth Bass (*Micropterus salmoides*) Fed a High-Starch Diet. *Frontiers in Marine Science*.

[77] Ahmadifar E., Moghadam M. S., Dawood M. A. O., Hoseinifar S. H. (2019). *Lactobacillus fermentum* and/or Ferulic Acid Improved the Immune Responses, Antioxidative Defence and Resistance Against *Aeromonas hydrophila* in Common Carp (*Cyprinus carpio*) Fingerlings. *Fish and Shellfish Immunology*.

[78] Hoseinifar S. H., Shakouri M., Doan H. V. (2020). Dietary Supplementation of Lemon Verbena (*Aloysia citrodora*) Improved Immunity, Immune-Related Genes Expression and Antioxidant Enzymes in Rainbow Trout (*Oncorrhyncus Mykiss*). *Fish and Shellfish Immunology*.

[79] Rafieepour A., Hajirezaee S., Rahimi R. (2020). Dietary Oregano Extract (*Origanum vulgare* L.) Enhances the Antioxidant Defence in Rainbow Trout, *Oncorhynchus mykiss* Against Toxicity Induced by Organophosphorus Pesticide, Diazinon. *Toxin Reviews*.

[80] Sönmez A. Y., Bilen S., Alak G., Hisar O., Yanık T., Biswas G. (2015). Growth Performance and Antioxidant Enzyme Activities in Rainbow Trout (*Oncorhynchus mykiss*) Juveniles Fed Diets Supplemented With Sage, Mint and Thyme Oils. *Fish Physiology and Biochemistry*.

[81] Raissy M., Ghafarifarsani H., Hoseinifar S. H., El-Haroun E. R., Naserabad S. S., Van Doan H. (2022). The Effect of Dietary Combined Herbs Extracts (oak Acorn, Coriander, and Common Mallow) on Growth, Digestive Enzymes, Antioxidant and Immune Response, and Resistance Against *Aeromonas hydrophila* Infection in Common Carp, *Cyprinus carpio*. *Aquaculture*.

[82] Ghafarifarsani H., Hoseinifar S. H., Sheikhlar A. (2022). The Effects of Dietary Thyme Oil (*Thymus vulgaris*) Essential Oils for Common Carp (*Cyprinus carpio*): Growth Performance, Digestive Enzyme Activity, Antioxidant Defense, Tissue and Mucus Immune Parameters, and Resistance Against *Aeromonas hydrophila*. *Aquaculture Nutrition*.

[83] Tan X., Sun Z., Liu Q. (2018). Effects of Dietary Ginkgo Biloba Leaf Extract on Growth Performance, Plasma Biochemical Parameters, Fish Composition, Immune Responses, Liver Histology, and Immune and Apoptosis-Related Genes Expression of Hybrid Grouper (*Epinephelus lanceolatus*♂ × *Epinephelus fuscoguttatus*♀) Fed High Lipid Diets. *Fish and Shellfish Immunology*.

[84] He G., Sun H., Liao R. (2022). Effects of Herbal Extracts (*Foeniculum vulgare* and *Artemisia annua*) on Growth, Liver Antioxidant Capacity, Intestinal Morphology and Microorganism of Juvenile Largemouth Bass, *Micropterus salmoides*. *Aquaculture Reports*.

[85] Abdel-Latif H. M. R., Ahmed H. A., Shukry M., Chaklader M. R., Saleh R. M., Khallaf M. A. (2022). *Astragalus Membranaceus* Extract (AME) Enhances Growth, Digestive Enzymes, Antioxidant Capacity, and Immunity of, *Pangasianodon hypophthalmus*, Juveniles. *Fishes*.

[86] Shohreh P., Mousavi S., Khoshbakht R. (2025). Immunostimulatory Effects of Mazari Palm (*Nannorrhops Ritchiana*) Leaves Extract on the Performance, Anti-Inflammation Genes, and Resistance of Grass Carp (*Ctenopharyngodon idella*) Juveniles to *Aeromonas hydrophila* Infection. *Animal Feed Science and Technology*.

[87] Shekarabi S. P. H., Mehrgan M. S., Ramezani F. (2022). Effect of Dietary Barberry Fruit (*Berberis vulgaris*) Extract on Immune Function, Antioxidant Capacity, Antibacterial Activity, and Stress-Related Gene Expression of Siberian Sturgeon (*Acipenser baerii*). *Aquaculture Reports*.

[88] Oh H. Y., Lee T. H., Lee D.-Y. (2022). Dietary Supplementation With Ginger (*Zingiber officinale*) Residue From Juice Extraction Improves Juvenile Black Rockfish (*Sebastes schlegelii*) Growth Performance, Antioxidant Enzyme Activity and Resistance to, *Streptococcus iniae*, Infection. *Animals*.

[89] Abdelhafiz Y., Gora A. H., Rehman S. (2023). Fish as the Lesser-Known Counterpart to Mammalian Models to Explore the Biofunctionality of Polyphenols. *Journal of Functional Foods*.

[90] Lv Q.-z., Long J.-t., Gong Z.-f. (2021). Current State of Knowledge on the Antioxidant Effects and Mechanisms of Action of Polyphenolic Compounds. *Natural Product Communications*.

[91] Kumar S., Pandey A. K. (2013). Chemistry and Biological Activities of Flavonoids: An Overview. *The Scientific World Journal*.

[92] Magaña A. A., Kamimura N., Soumyanath A., Stevens J. F., Maier C. S. (2021). Caffeoylquinic Acids: Chemistry, Biosynthesis, Occurrence, Analytical Challenges, and Bioactivity. *The Plant Journal*.

[93] Li X., Li K., Xie H. (2018). Antioxidant and Cytoprotective Effects of the Di-O-Caffeoylquinic Acid Family: The Mechanism, Structure-Activity Relationship, and Conformational Effect. *Molecules*.

[94] Liu W., Li J., Zhang X. (2020). Current Advances in Naturally Occurring Caffeoylquinic Acids: Structure, Bioactivity, and Synthesis. *Journal of Agricultural and Food Chemistry*.

[95] Bešlo D., Golubić N., Rastija V. (1141). Antioxidant Activity, Metabolism, and Bioavailability of Polyphenols in the Diet of Animals. *Antioxidants*.

[96] Méndez L., Medina I. (2021). Polyphenols and Fish Oils for Improving Metabolic Health: A Revision of the Recent Evidence for Their Combined Nutraceutical Effects. *Molecules*.

[97] Xiao P., Ji H., Ye Y. (2017). Dietary Silymarin Supplementation Promotes Growth Performance and Improves Lipid Metabolism and Health Status in Grass Carp (*Ctenopharyngodon Idellus*) Fed Diets with Elevated Lipid Levels. *Fish Physiology and Biochemistry*.

[98] Hanhineva K., Törrönen R., Bondia-Pons I. (2010). Impact of Dietary Polyphenols on Carbohydrate Metabolism. *International Journal of Molecular Sciences*.

[99] Li S., Tan H. Y., Wang N., Cheung F., Hong M., Feng Y. (2018). The Potential and Action Mechanism of Polyphenols in the Treatment of Liver Diseases. *Oxidative Medicine and Cellular Longevity*.

[100] Mokhtar D. M., Zaccone G., Alesci A., Kuciel M., Hussein M. T., Sayed R. K. A. (2023). Main Components of Fish Immunity: An Overview of the Fish Immune System. *Fishes*.

[101] Firmino J. P., Galindo-Villegas J., Reyes-López F. E., Gisbert E. (2021). Phytogenic Bioactive Compounds Shape Fish Mucosal Immunity. *Frontiers in Immunology*.

[102] Elumalai P., Kurian A., Lakshmi S., Faggio C., Esteban M. A., Ringø E. (2021). Herbal Immunomodulators in Aquaculture. *Reviews in Fisheries Science and Aquaculture*.

[103] Joibari F. G., Bahrekazemi M., Keshavarz M., Bahram S., Javadian S. R., Abdel-Tawwab M. (2025). Stimulatory Effects of Dietary Savory (*Satureja hortensis* L.) Ethanolic Extract on Growth, Digestive Enzymes, Immune Parameters, Antioxidant Defense, and Immune and Antioxidant-Related Genes in Zebrafish (*Danio rerio*). *Animal Feed Science and Technology*.

[104] Saurabh S., Sahoo P. (2008). Lysozyme: An Important Defence Molecule of Fish Innate Immune System. *Aquaculture Research*.

[105] Coeurdacier J.-L., Dutto G., Gasset E., Blancheton J.-P. (2011). Is Total Serum Protein a Good Indicator for Welfare in Reared Sea Bass (*Dicentrarchus labrax*)?. *Aquatic Living Resources*.

[106] Adel M., Dawood M. A. O., Gholamhosseini A., Sakhaie F., Banaee M. (2021). Effect of the Extract of Lemon Verbena (*Aloysia citrodora*) on the Growth Performance, Digestive Enzyme Activities, and Immune-Related Genes in Siberian Sturgeon (*Acipenser baerii*). *Aquaculture*.

[107] Moghanlou K. Sarvi, Isfahani E. Nasr, Dorafshan S., Tukmechi A., Aramli M. S. (2018). Effects of Dietary Supplementation With, *Stachys Lavandulifolia*, Vahl Extract on Growth Performance, Hemato-Biochemical and Innate Immunity Parameters of Rainbow Trout (*Oncorhynchus mykiss*). *Animal Feed Science and Technology*.

[108] Sendijani R. Z., Kenari A. A., Smiley A. H., Esmaeili N. (2020). The Effect of Extract from Dill *Anethum graveolens* on the Growth Performance, Body Composition, Immune System, and Antioxidant System of Rainbow Trout. *North American Journal of Aquaculture*.

[109] Rashidian G., Kajbaf K., Prokić M. D., Faggio C. (2020). Extract of Common Mallow (*Malvae Sylvestris*) Enhances Growth, Immunity, and Resistance of Rainbow Trout (*Oncorhynchus mykiss*) Fingerlings Against *Yersinia ruckeri* Infection. *Fish and Shellfish Immunology*.

[110] Ghafarifarsani H., Yousefi M., Hoseinifar S. H. (2022). Beneficial Effects of Persian Shallot (*Allium Hirtifolium*) Extract on Growth Performance, Biochemical, Immunological and Antioxidant Responses of Rainbow Trout *Oncorhynchus mykiss* Fingerlings. *Aquaculture*.

[111] Ahmadi K., Banaee M., Vosoghei A. R., Mirvaghefei A. R., Ataeimehr B. (2012). Evaluation of the Immunomodulatory Effects of Silymarin Extract (*Silybum marianum*) on Some Immune Parameters of Rainbow Trout, *Oncorhynchus mykiss* (Actinopterygii: Salmoniformes: Salmonidae). *Acta Ichthyologica Et Piscatoria*.

[112] Wang J., Zhou H., Wang X., Mai K., He G. (2019). Effects of Silymarin on Growth Performance, Antioxidant Capacity and Immune Response in Turbot (*Scophthalmus maximus* L.). *Journal of the World Aquaculture Society*.

[113] Rohmah M. K., Salahdin O. D., Gupta R. (2022). Modulatory Role of Dietary Curcumin and Resveratrol on Growth Performance, Serum Immunity Responses, Mucus Enzymes Activity, Antioxidant Capacity and Serum and Mucus Biochemicals in the Common Carp, *Cyprinus carpio* Exposed to Abamectin. *Fish and Shellfish Immunology*.

[114] Zhang R., Liu L. L., Wang X. W., Guo C. Y., Zhu H. (2020). Dietary Tea Polyphenols Induce Changes in Immune Response and Intestinal Microbiota in Koi Carp, *Cyprinus carpio*. *Aquaculture*.

[115] Harikrishnan R., Balasundaram C., Heo M.-S. (2011). Impact of Plant Products on Innate and Adaptive Immune System of Cultured Finfish and Shellfish. *Aquaculture*.

[116] Zhang W., Zhao J., Ma Y., Li J., Chen X. (2022). The Effective Components of Herbal Medicines Used for Prevention and Control of Fish Diseases. *Fish and Shellfish Immunology*.

[117] Nootash S., Sheikhzadeh N., Baradaran B. (2013). Green Tea (*Camellia sinensis*) Administration Induces Expression of Immune Relevant Genes and Biochemical Parameters in Rainbow Trout (*Oncorhynchus mykiss*). *Fish and Shellfish Immunology*.

[118] Mehrabi Z., Firouzbakhsh F., Rahimi-Mianji G., Paknejad H. (2020). Immunity and Growth Improvement of Rainbow Trout (*Oncorhynchus mykiss*) Fed Dietary Nettle (*Urtica dioica*) Against Experimental Challenge With *Saprolegnia parasitica*. *Fish and Shellfish Immunology*.

[119] Baba E., Acar Ü., Yılmaz S., Zemheri F., Ergün S. (2018). Dietary Olive Leaf (*Olea Europea* L.) Extract Alters Some Immune Gene Expression Levels and Disease Resistance to *Yersinia ruckeri* Infection in Rainbow Trout *Oncorhynchus mykiss*. *Fish and Shellfish Immunology*.

[120] Wang Q., Shen J., Yan Z. (2020). Dietary *Glycyrrhiza uralensis* Extracts Supplementation Elevated Growth Performance, Immune Responses and Disease Resistance Against *Flavobacterium columnare* in Yellow Catfish (*Pelteobagrus Fulvidraco*). *Fish and Shellfish Immunology*.

[121] Secombes C. J., Wang T., Hong S. (2001). Cytokines and Innate Immunity of Fish. *Developmental and Comparative Immunology*.

[122] Sakai M., Hikima J., Kono T. (2021). Fish Cytokines: Current Research and Applications. *Fisheries Science*.

[123] Awad E., Mitchell W. J., Austin B. (2011). Effect of Dietary Supplements on Cytokine Gene Expression in Rainbow Trout, *Oncorhynchus mykiss* (Walbaum). *Journal of Fish Diseases*.

[124] Sebastián R.-C., Kevin M., Felipe R.-L., Daniela T.-A., María S. Ana, Mónica I., Hakan T. (2012). Fish Cytokines and Immune Response. *New Advances and Contributions to Fish Biology*.

[125] Magrone T., Spagnoletta A., Magrone M. (2019). Effects of Polyphenol Administration to European Farmed Sea Bass (*Dicentrharcus Labrax* L.): Special Focus on Hepatopancreas Morphology. *Endocrine, Metabolic and Immune Disorders - Drug Targets*.

[126] Cirmi S., Randazzo B., Russo C. (2021). Anti-Inflammatory Effect of a Flavonoid-Rich Extract of Orange Juice in Adult Zebrafish Subjected to *Vibrio anguillarum*-Induced Enteritis. *Natural Product Research*.

